# 

**Published:** 1870-06

**Authors:** 


*Harris' Principles and Practice of Dental Surgery.*—This work is now being revised by Drs. Austen, Latimer and Gorgas, of the Baltimore College of Dental Surgery, and it is expected that the new edition will be issued by the Publishers, Lindsay and Blakiston, of Philadelphia, early in the coming fall.

